# Berry Extracts and Their Bioactive Compounds Mitigate LPS and DNFB-Mediated Dendritic Cell Activation and Induction of Antigen Specific T-Cell Effector Responses

**DOI:** 10.3390/antiox12091667

**Published:** 2023-08-24

**Authors:** Puja Upadhaya, Felipe F. Lamenza, Suvekshya Shrestha, Peyton Roth, Sushmitha Jagadeesha, Hasan Pracha, Natalie A. Horn, Steve Oghumu

**Affiliations:** 1 Department of Pathology, The Ohio State University Wexner Medical Center, Columbus, OH 43210, USA; upadhaya.1@osu.edu (P.U.); lamenza.1@buckeyemail.osu.edu (F.F.L.); shrestha.119@buckeyemail.osu.edu (S.S.); roth.633@buckeyemail.osu.edu (P.R.); jagadeesha.2@buckeyemail.osu.edu (S.J.); pracha.3@buckeyemail.osu.edu (H.P.); horn.420@buckeyemail.osu.edu (N.A.H.); 2 Department of Microbiology, The Ohio State University, Columbus, OH 43210, USA

**Keywords:** contact hypersensitivity, berries, antioxidants, immune response

## Abstract

Berries have gained widespread recognition for their abundant natural antioxidant, anti-inflammatory, and immunomodulatory properties. However, there has been limited research conducted thus far to investigate the role of the active constituents of berries in alleviating contact hypersensitivity (CHS), the most prevalent occupational dermatological disease. Our study involved an ex vivo investigation aimed at evaluating the impact of black raspberry extract (BRB-E) and various natural compounds found in berries, such as protocatechuic acid (PCA), proanthocyanidins (PANT), ellagic acid (EA), and kaempferol (KMP), on mitigating the pathogenicity of CHS. We examined the efficacy of these natural compounds on the activation of dendritic cells (DCs) triggered by 2,4-dinitrofluorobenzene (DNFB) and lipopolysaccharide (LPS). Specifically, we measured the expression of activation markers CD40, CD80, CD83, and CD86 and the production of proinflammatory cytokines, including Interleukin (IL)-12, IL-6, TNF-α, and IL-10, to gain further insights. Potential mechanisms through which these phytochemicals could alleviate CHS were also investigated by investigating the role of phospho-ERK. Subsequently, DCs were co-cultured with T-cells specific to the OVA_323-339_ peptide to examine the specific T-cell effector responses resulting from these interactions. Our findings demonstrated that BRB-E, PCA, PANT, and EA, but not KMP, inhibited phosphorylation of ERK in LPS-activated DCs. At higher doses, EA significantly reduced expression of all the activation markers studied in DNFB- and LPS-stimulated DCs. All compounds tested reduced the level of IL-6 in DNFB-stimulated DCs in Flt3L as well as in GM-CSF-derived DCs. However, levels of IL-12 were reduced by all the tested compounds in LPS-stimulated Flt3L-derived BMDCs. PCA, PANT, EA, and KMP inhibited the activated DC-mediated Interferon (IFN)-γ and IL-17 production by T-cells. Interestingly, PANT, EA, and KMP significantly reduced T-cell proliferation and the associated IL-2 production. Our study provides evidence for differential effects of berry extracts and natural compounds on DNFB and LPS-activated DCs revealing potential novel approaches for mitigating CHS.

## 1. Introduction

Contact hypersensitivity (CHS) is a type 4 delayed-type hypersensitivity reaction, triggered by an immune response that occurs when the skin comes into contact with an allergen or a hapten [1]. CHS is a prevalent occupational dermatological condition, affecting approximately 15–20% of the general adult population. It is frequently caused by fragrant cosmetics, with stabilizing and texture agents being the two most significant allergens found in these products [2,3,4,5]. Additional triggers include a variety of workplace and lifestyle items like metals, detergents, rubber gloves, jewelry, and dyes [6,7,8]. CHS is a common cause of allergic contact dermatitis (ACD), a skin condition which manifests in patients as itching, burning pain, and general discomfort mediated by an antigen-specific T-cell response [9]. These symptoms can have a negative impact on patients’ mood, behavior, and sleep, ultimately leading to a reduced quality of life [10]. CHS is characterized as a dendritic cell-dependent, T-cell-derived, cytokine-mediated skin inflammation that undergoes two distinct progression phases: (1) sensitization phase and (2) elicitation phase [11]. During the sensitization phase, an allergen or hapten activates the innate immune system and delivers signals that enable the recruitment, migration, and maturation of dendritic cells (DCs), which are specialized antigen presenting cells and the mediators of adaptive immunity [12,13,14]. These allergens or haptens are engulfed and processed within DCs and are expressed in MHC class I and class II molecules [15], leading to DC maturation and migration to regional lymph nodes [16]. During this maturation process they undergo several metabolic and phenotypic alterations [17,18], including secretion of the cytokines interleukin (IL) 1β, IL-6, IL-12, or tumor necrosis factor (TNF) α and the upregulation of adhesion molecules, chemokine receptors, and co-stimulatory molecules [19].

Molecular and immunologic mechanisms underlying allergen-specific DC-mediated alterations are still not completely understood, but they are essential for effective treatment strategies against CHS. Signaling mediators and cytokines that have been found to be altered in DCs include nuclear factor NF-kappa-B p105 subunit (NFKB1), nuclear factor NF-kappa-B p100 subunit (NFKB2), TNF receptor-associated factor (TRAF) 1, E3 ubiquitin-protein ligase TRIM4, CD54, fascin, signal transducer and activator of transcription (STAT), IL-1β, IL-9, and interferon and their associated signaling pathways [20]. Furthermore, previous studies with human donor-derived peripheral blood CD14+ monocytes (Mo-DC) when cultured with 2,4-dinitrofluorobenzene (DNFB) or NiSO4 (contact allergens) demonstrated an increase phosphorylation of p38 MAPK and ERK indicating that these pathways play an active role in the maturation of DCs induced by allergens [21,22,23]. Recent studies have found that the presence of a contact allergen triggers the activation of sensory nerves, facilitating the migration of DCs to the lymph nodes, thereby promoting effective priming of T-cells [24]. At regional lymph nodes, mature DCs prime allergen specific T-cells leading to their activation, proliferation, and subsequent emigration to peripheral sites of allergen exposure [25]. During the elicitation phase, allergen specific T-cells elicit a type 1 inflammatory immune response mediated by macrophage activation and cytokine production, which drives the pathogenesis of CHS [26]. Key cytokines which mediate CHS include IFN-α produced by CD8+ T-cells and IFN-γ produced by Th1 cells and CD8+ T-cells [25,27,28,29,30]. Polarization of CD8+ T-cells to a type 1 phenotype is essential for CHS development, while polarization to a type 2 phenotype reduces the responses [31,32]. IL-17 potentially amplifies the allergic reaction by rendering T-cell populations capable of contributing to tissue damage at the site of inflammation [33,34,35].

Therapeutic approaches for CHS currently involve avoiding irritants and allergens, utilizing emollients, and administering topical glucocorticosteroids [36]. However, these treatments often have limitations and carry the risk of potential side effects, including immune deficiency, weight gain, localized skin atrophy, and osteoporosis [37,38,39]. Hence, it is imperative to explore alternative approaches for the treatment of CHS. Ideally, the therapeutic approach should have the ability to influence allergic sensitization and provide relief from allergic symptoms after sensitization to the allergen. Additionally, the treatment should demonstrate effectiveness against various allergic disorders and maintain long-term safety during administration. 

Natural products have been widely utilized for therapeutic purposes, and many of these bioactive compounds have been integrated into modern pharmaceutical drugs and medicinal products [40]. Much of their health benefits are attributed to phytochemicals or secondary metabolites as they provide unique structural diversity, presenting opportunities as novel drug candidates [40,41,42]. Berries have been shown to demonstrate significant health benefits due to their antioxidant and anti-inflammatory properties and their rich source of bioactive phytochemicals [43]. Berries are a good source of natural antioxidant substances such as anthocyanins, flavonoids, and phenolic acids and have high antioxidant enzymes and oxygen radical scavenging activities [44]. 

Black raspberries (BRB; *Rubus occidentalis*) have a profound role to play in promoting good health and in the treatment of diseases, including chronic inflammation and cancers [45,46,47,48,49,50,51,52]. BRBs are abundant in anthocyanins, which possess anti-inflammatory and antioxidant properties, providing protective benefits [45]. Additionally, BRBs contain a diverse range of bioactive phytochemicals, including phytosterols like β-sitosterol, various polyphenols such as ellagic acid, ferulic acid, ellagitannins, anthocyanins, protocatechuic acid (PCA), and bioflavonoids like quercetin. These compounds collectively contribute to the positive impact of BRBs on overall health [53,54,55]. Consumption of BRBs at 35–45 g/day has been shown to reduce oxidative stress in patients [56].

Our group previously demonstrated the efficacy of BRBs in mitigating contact dermatitis in a murine model of CHS using DNFB [50]. Although a majority of the anti-inflammatory and antioxidant properties of BRBs are attributable to the synergistic combination of bioactive phytochemicals, several individual berry phytochemicals and metabolites demonstrate anti-inflammatory and antioxidant properties. Proanthocyanidins (PANT) are bioactive polyphenolic compounds that occur naturally in plants, including berries and various other plant sources [57,58]. In BRBs, PANTs are found at a concentration ranging between 3–74 mg/100 g [58]; PANTs have the potential to alleviate oxidative stress and inflammation. Additionally, they have been shown to ameliorate allergic contact dermatitis or CHS by inhibiting the activation of T-cells [59]. Ellagic acid (EA) is a bioactive phytochemical naturally present in berries that is metabolized to produce urolithins, which are compounds known for their potent anti-inflammatory, antioxidant, immunomodulatory, and neuroprotective properties [60,61]. EA is found in significant concentrations in BRBs and strawberries [62]. BRBs contain EA at a concentration of about 3.7–3.9 mg/100 g of the fruit [63]. One notable characteristic of EA is its predicted regenerative capacity, enabling it to offer protection against oxidants even at low concentrations [64,65,66]. EA’s anti-inflammatory and antioxidant properties are evidenced by a reduction in pro-inflammatory cytokines and normalized levels of antioxidant enzymes [67,68,69,70]. Published reports have documented the role of EA in alleviating pruritus (itching) associated with allergic contact dermatitis [64,65]. Kaempferol (KMP) has been studied for its anti-inflammatory properties through various mechanisms such as the inhibition of T-cell proliferation or DC activation [71]. A study conducted by Häkkinen et al. examined 25 types of edible berries, and found that KMP was present only in gooseberries (with concentrations of 16 and 19 mg/kg) and strawberries (with concentrations of 5 and 8 mg/kg) [72]. Studies have shown that KMP increases ROS-dependent apoptosis in pancreatic tumor cells through TGM2-mediated Akt/mTOR signaling [73]. KMP is associated with ameliorating atopic dermatitis [74] and immediate-type hypersensitivity [75,76]. Protocatechuic acid (PCA) is a gut bacterial metabolite of BRB [77]. It is most prevalent among various small and easily absorbed gut microbial metabolites derived from anthocyanins, which constitutes more than 70% of the anthocyanin metabolites from berries in the human body. In BRBs, the amount of PCA is around 8.35 mg/100 g dry weight [78]. PCA has been identified as a potential catalyst for the immunomodulatory effects exerted by berries [75,76]. It has abundant pharmacological activities including antioxidant, neuroprotective, antibacterial, protection of metabolic syndrome, and preservation of various organ functions [79]. 

Although BRB and major bioactive constituents have been demonstrated to ameliorate the effects of contact hypersensitivity, the underlying molecular and immunological mechanisms are still not completely characterized [50]. Therefore, we determined the mechanisms underlying the efficacy of a BRB extract and plant polyphenols present in berries to mitigate CHS with a focus on dendritic cell activation and induction of antigen-specific T-cell responses. To do this, we employed an ex vivo experimental model using (i) activation of bone marrow-derived DCs, which is crucial for mediating the sensitization phase of CHS in an in vivo setting.(ii) Using ovalbumin transgenic (OT-II) mice, which carry a T-cell receptor specific to an allergen (OVA_323–339_ peptide), we examined the specific T-cell effector responses induced by activated DCs exposed to BRB extract or individual bioactive compounds. Additionally, we aimed to explore the potential mechanisms through which these phytochemicals could alleviate the pathogenicity associated with CHS. Our findings provide evidence for differential effects of berry extracts and natural compounds on the alleviation of CHS pathology, revealing potential novel approaches for mitigating CHS.

## 2. Materials and Methods

### 2.1. Animal Handling

Six-week-old C57BL/6 and OT-II TCR transgenic mice were purchased from Jackson Laboratories (Bar Harbor, MI, USA). The mice were kept in a controlled environment with a 12-h light/dark cycle and provided with food and water ad libitum. The animal care and use procedures were in compliance with the regulations set by the university’s Laboratory Animal Resources and were approved by the Institutional Animal Care and Use Committee (Protocol#2018A00000054) and Institutional Biosafety Committee of The Ohio State University.

### 2.2. Black Raspberry Extract Preparation for the Study

Black raspberry powder and preparation of extracts was performed as described previously [50]. Briefly, BRB powder was extracted three times with 80:19:1 ethanol/water/formic acid. The mixture was sonicated for 10 min in an ice bath and then vacuum filtered. The resulting extract was concentrated using a rotary evaporator and further subjected to lyophilization to eliminate any residual water. This procedure yielded 40 g of extract per 100 g of BRB powder, with an anthocyanin content of 57 milligrams of cyanidin-3-glucoside equivalents per gram of extract. The analysis of anthocyanins in the BRB extract followed previously described protocols [80].

### 2.3. Natural Compounds

Protocatechuic acid (PCA; Catalogue no. 114895000) and Ellagic acid (EA; Catalogue no. 117740500) was purchased from Acros Organics, NJ, USA. Proanthocyanidins (PANT; Catalogue no. HY-N0794) was purchased from Med Chem Express (MCE), NJ, USA, and Kaempferol (KMP; Catalogue no. 11852) was purchased from Cayman Chemical Company, Ann Arbor, MI, USA.

### 2.4. Generation of Bone Marrow-Derived DCs by GM-CSF and Flt3 Ligand and Stimulation

To isolate dendritic cells, bone marrow cells were collected from euthanized C57BL/6 mice by harvesting tibias and femurs. The cells were then treated with ACK lysis buffer and cultured in complete RPMI medium supplemented with 10% fetal bovine serum (Atlanta Biologicals, Flowery Branch, GA, USA), 1% penicillin (20 Units/mL)/streptomycin (20 µg/mL) (Life Technologies, Carlsbad, CA, USA), and 25 ng/mL recombinant mouse granulocyte macrophage colony-stimulating factor (GM-CSF)/Flt3 Ligand for 7 days. Generation of DCs from Flt3 Ligand was performed following a protocol by Lutz et al. [81]. Cells were then plated in 12-well plates at a concentration of 2 × 10^5^ cells/well for GM-CSF-derived DCs and in 24-well plates at a concentration of 7 × 10^4^ cells/well for Flt3 Ligand-derived DCs. DCs were randomly divided into groups receiving no treatment or receiving one of the following treatments: Black Raspberry Extract (BRB-E, at concentrations of 5 µg/mL or 50 µg/mL), Protocatechuic acid (PCA, at concentrations of 5 and 50 µM), Proanthocyanidins (PANT, at concentrations of 5 and 50 µM), Ellagic acid (EA, at concentrations of 5 and 50 µM), and Kaempferol (KMP, at concentrations of 5 and 50 µM) dissolved in DMSO in a total volume of 500 µL/well. To conduct the activation studies, after 24 h treatment with compounds, some samples in each group were stimulated with 100 ng/mL of lipopolysaccharide (LPS) for 6 h and 24 h, and 5 µg/mL of DNFB for 18 h alongside the respective treatment groups. After 6 h of LPS stimulation, cell lysates were collected for protein analysis using Western Blotting. For treatments lasting 24 h for LPS and 18 h for DNFB, cells were collected for flow cytometric analysis of activation markers and supernatants were collected for cytokine ELISA.

### 2.5. Western Blotting

DCs were exposed to either DMSO or varying concentrations of natural compounds, followed by immediate stimulation with LPS at a concentration of 100 ng/mL for a duration of 6 h. Afterward, the cells were harvested and 20 μg of protein was loaded onto a 10% Tris-HCL gel. The proteins were then transferred to a PVDF membrane (0.2 µm). The membrane blots were subsequently blocked using 5% non-fat dry milk for 1 h and incubated with the primary antibody rabbit anti-mouse phospho-p44/42 MAPK (Erk1/2) (Cell Signaling, Danvers, MA, USA, 4370S), p44/42 MAPK (Erk1/2) (Cell Signaling, 4695S), and Rabbit GAPDH (Cell Signaling, 2118S) overnight. Blots were incubated with goat anti-rabbit HRP-linked secondary antibody (31460, Thermo Fisher Scientific, Rockland, IL, USA) for 1 h. Chemiluminescense was detected by ECL western blotting substrate (Thermo Scientific, Waltman, MA, USA). Image J, version 1.8.0 was used to quantify the protein intensity.

### 2.6. Enzyme-Linked Immunosorbent Assay

Supernatants were collected from DCs treated with different doses of natural compounds for 24 h. The production of cytokines IL-12, IL-6, TNF-α, and 1L-10 was measured using ELISA. Supernatants from DCs co-cultured with OT-II T-cells were collected and assayed for IFN-γ, IL-17, and IL-2. All the capture and detection antibodies were purchased from BioLegend (San Diego, CA, USA).

### 2.7. T-Cell Activation and Proliferation

Ag presentation by DCs was determined by using the OVA specific T-cell proliferation ex vivo assay. DCs were seeded in 24-well plates at a concentration of 2 × 10^5^ cells/well and pulsed with OVA_323–339_ peptide (2 µg/mL) and incubated with LPS (100 ng/mL), and higher doses of the natural compounds, BRB-E (50 µg/mL), PCA (50 µM), PANT (50 µM), EA (50 µM), and KMP (50 µM). Cells were then washed with media to remove the natural compounds. T-cells were isolated from the OT-II TCR transgenic mice using the EasySep Mouse T-cell isolation Kit (STEMCELL Technologies, Vancouver, BC, Canada; 19851) and stained with 1 µM CFSE. Isolated T-cells were washed and added to the DC cultures and subsequently incubated for 72 h. T-cell proliferation was determined by flow cytometry.

### 2.8. Flow Cytometry

Flow cytometric assay was performed to determine the cell surface expression of activation markers CD40, CD80, CD83, and CD86 on -LPS/DNFB-activated DCs. Cells were incubated with fluorochrome conjugated antibodies targeting CD11b, CD11c, CD40, CD80, CD83, and CD86 (BioLegend, San Jose, CA, USA). Cells were also stained with PI to check for cell viability. For T-cell proliferation studies, DCs co-cultured with T-cells were incubated with fluorochrome conjugated antibodies targeting CD4, CD8, and intracellularly stained for IFN-γ, and Perforin (BioLegend, San Jose, CA, USA). Samples were analyzed using a FACS Celesta flow cytometer (BD Biosciences, San Jose, CA, USA). All flow cytometric data analyses were conducted using the FlowJo software, version 10.8.1 (Tree Star Inc., Ashland, OR, USA).

### 2.9. Statistical Analysis

Statistical analyses were conducted using GraphPad Prism software v9.2.0 (GraphPad Software, San Diego, CA, USA). Ordinary one-way Analysis of variance (ANOVA) with Dunnett’s multiple comparisons test was employed to assess the statistical significance of differences between groups, with a *p*-value threshold of 0.05.

## 3. Results and Discussion

### 3.1. Effects of BRB-E, Protocatechuic Acid, Proanthocyanidins, Ellagic Acid and Kaempferol on the Activation of ERK in GM-CSF-Derived LPS-Stimulated DCs

**Figure 1:**
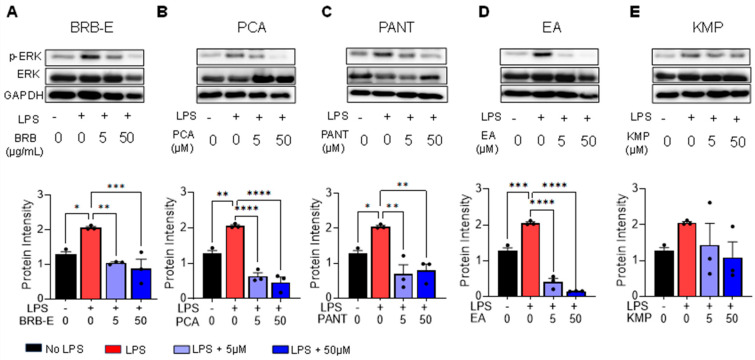
Expression of p-ERK and ERK in LPS-stimulated, BRB-E, protocatechuic acid, proanthocyanidins, ellagic acid, and kaempferol pretreated BMDCs. (**A**–**E**) Representative western blot images of p-ERK, ERK, and GAPDH protein levels in either LPS non-stimulated or LPS-stimulated DCs pretreated with BRB-E, PCA, PANT, EA, and KMP. Cell lysates were collected from DCs after 6 h LPS (100 ng/mL) stimulation. Average protein intensity of western blot bands was measured for different groups using ImageJ. (**A**–**E**) Decreased protein intensity of p-ERK in DCs after treatment with (**A**) BRB-E, (**B**) PCA, (**C**) PANT, and (**D**) EA. Data are represented as mean ± SEM relative to the GAPDH control (N = 3 per group). * *p*-value < 0.05; ** *p*-value < 0.01; *** *p*-value < 0.001; **** *p*-value < 0.0001 for comparisons between the LPS to no LPS and the natural compound treatment groups using one-way ANOVA.

Dendritic cells are principal regulators of the innate and adaptive immune response and are responsible for initiating responses to allergens during CHS [82]. We employed an in vitro model using LPS stimulation of DCs, which recapitulates the molecular events of DC activation during CHS to determine the effects of BRB-E and individual berry bioactive compounds PCA, PANT, EA, and KMP on DC activation. LPS induces phosphorylation of extracellular signal-regulated kinases (ERK), which is essential in regulating cellular proliferation, differentiation, maturation, and DC survival [83,84,85]. We therefore determined the effects of these berry compounds on ERK phosphorylation in BMDCs. To examine this, DCs were subjected to different concentrations (5 µg/mL and 50 µg/mL for BRB-E, and 5 µM and 50 µM for PCA, PANT, EA, and KMP) of natural compounds, then stimulated with LPS (100 ng/mL). Levels of phospho-ERK (p-ERK), total ERK (ERK), and GAPDH were measured by Western Blot. As expected, LPS stimulation of BMDCs resulted in increased p-ERK levels (Figure 1A–E). Further, LPS-induced activation of p-ERK was significantly decreased by the treatment with BRB-E, PCA, and EA in a dose dependent manner (Figure 1A,B,D). PANT also showed reduced p-ERK levels, but this reduction was not dose dependent (Figure 1C). Treatment with KMP did not result in a significant reduction in p-ERK levels (Figure 1E). No changes were observed in the total ERK levels in any of the treatment groups. Taken together, our results suggest that BRB-E, PCA, EA, and PANT inhibits ERK phosphorylation in LPS-activated DCs.

Previous reports have found that phosphorylation of ERK occurs in DCs when incubated with DNFB or NiSO4 [21,22,23]. Furthermore, inhibition of ERK pathways were found to cause a selective inhibition of CD86, CD54, and/or CD40, as well as TNF-α expression, indicating a delay in DC maturation [86]. It is therefore likely that inhibition of ERK phosphorylation is a key mechanism by which BRB-E, PCA, PANT, and EA suppress DC activation during CHS. We previously demonstrated BRB-E inhibited CD80 expression in DCs in a murine model of CHS using DNFB [50]. Interestingly, application of topical ERK inhibitors has been shown to result in improved skin barrier function, reduced inflammatory cell infiltration, and alleviated dermatitis symptoms [87]. Similarly, a natural flavonoid, quercetin, found in fruits and vegetables, was demonstrated to inhibit DNFB-induced CHS through an inhibitory effect on the ERK pathway in activated DCs [88]. 

### 3.2. Effects of BRB-E, Protocatechuic Acid, Proanthocyanidins, Ellagic Acid, and Kaempferol on DNFB/- LPS-Stimulated DCs Activation Markers

**Figure 2:**
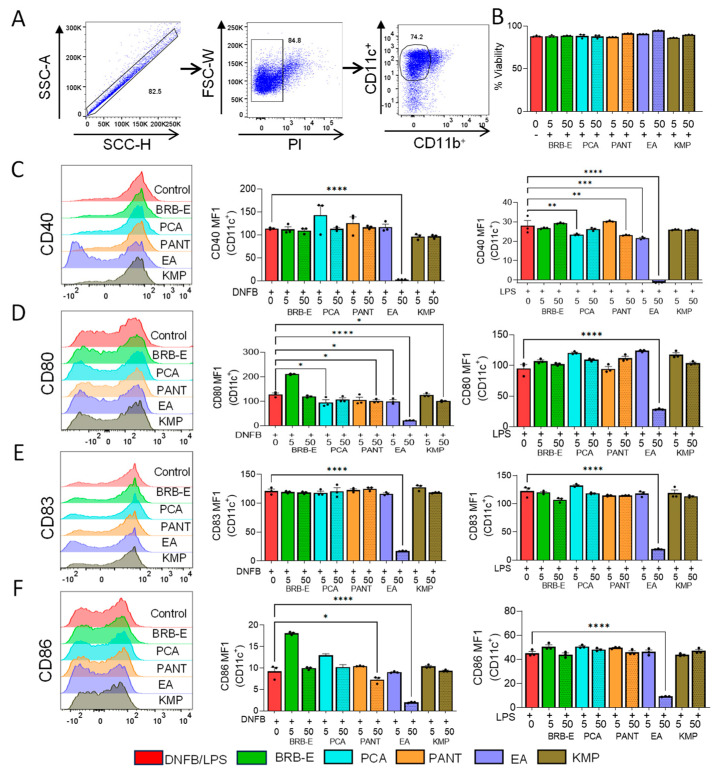
Effects of BRB-E, protocatechuic acid, proanthocyanidins, ellagic acid, and kaempferol on DNFB- and LPS-stimulated DC activation markers in Flt3L-derived BMDCs. (**A**) Gating strategy utilized to identify CD11b**^+^** and CD11c**^+^** cells. (**B**) Percent viability of DCs after treatment with natural compounds measured by propidium iodide staining. (**C**–**F**) Histograms and Mean fluorescent intensity (MFI) showing the population of cells positive for activation markers (**C**) CD40, (**D**) CD80, (**E**) CD83, and (**F**) CD86 expression by CD11c+ dendritic cells stimulated with DNFB and LPS. Solid lines represent the lower doses and shaded lines represent higher doses for each compound. Data are represented as mean ± SEM. * *p*-value < 0.05; ** *p*-value < 0.01; *** *p*-value < 0.001; **** *p*-value < 0.0001 for comparisons between the DNFB/LPS and the natural compound treatment groups using one-way ANOVA.

During CHS, dendritic cells play a crucial role by taking up antigens, migrating to secondary lymphoid organs, and acquiring antigen-presenting capabilities. This is evidenced by the expression of co-stimulatory molecules like CD80 and CD86 and activation markers CD40 and CD83. We investigated the effect of BRB-E, PCA, PANT, EA, and KMP on the expression of co-stimulatory molecules and activation markers in Flt3L-derived DNFB/-LPS-stimulated DCs using flow cytometry (Figure 2A). The viability of the cells following treatment with different compounds was determined by flow cytometry (Figure 2B). Upon subjecting the DCs to treatment with these compounds, we made several noteworthy observations. EA, when administered at higher doses (50 µM), led to a significant reduction in CD40 expression in both DNFB/-LPS-stimulated DCs (Figure 2C). Notably, in LPS-stimulated DCs, a dose-dependent decrease in CD40 expression was observed with increasing doses of EA (Figure 2C). Similarly, lower doses of PCA (5 µM) and higher doses of PANT (50 µM) also resulted in reduced CD40 expression (Figure 2C). 

We then examined CD80 expression and found a similar trend to that of CD40 with EA treatment. DNFB-stimulated DCs showed a dose-dependent decrease in CD80 expression upon EA treatment (Figure 2D), whereas LPS-stimulated DCs showed a significant reduction in CD80 expression at higher EA doses (Figure 2D). Additionally, KMP and PANT at higher doses, as well as PCA at lower doses, exhibited reduced CD80 expression in the DNFB-stimulated group (Figure 2D). Subsequently, we assessed the expression of CD83 and CD86. EA at higher doses demonstrated downregulation of both CD83 and CD86 expression in both LPS- and DNFB-stimulated DCs (Figure 2E,F). Moreover, PANT at higher doses (50 µM) was effective in reducing CD86 expression in DNFB-treated DCs (Figure 2). To ensure that the dendritic cell cultures were responding to DNFB and LPS stimulation, we included an activation cocktail control proposed by Hoyer et al. [89] in our study as a positive control and compared that to our DNFB and LPS control (Figure S1A,B). In summary, our activation studies revealed that all the natural compounds exerted some inhibitory effects on specific DC co-stimulatory and activation markers. However, EA at higher doses consistently displayed the greatest inhibition compared to other treatment groups across various activation markers.

### 3.3. Effects of BRB-E, Protocatechuic Acid, Proanthocyanidins, Ellagic Acid, and Kaempferol on the Production of IL-12 by Stimulated DCs

**Figure 3:**
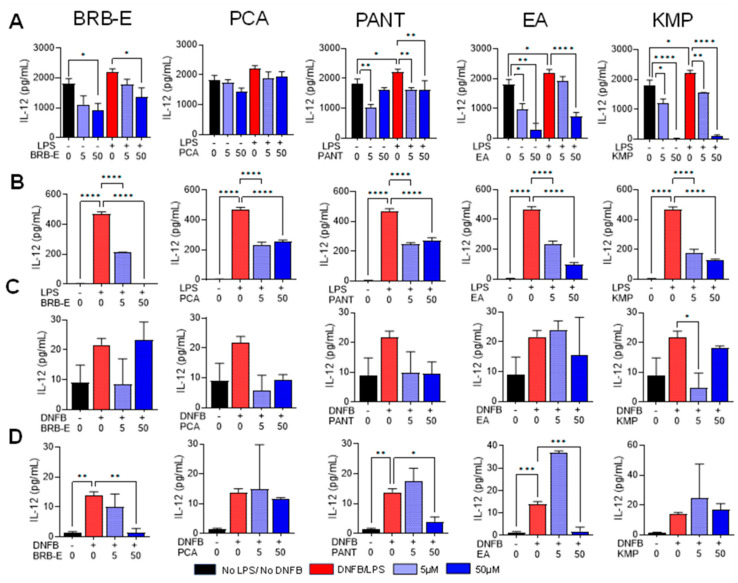
Effects of BRB-E, protocatechuic acid, proanthocyanidins, ellagic acid, and kaempferol on the production of IL-12 by stimulated DCs. Supernatants were collected from DCs after 24 h of LPS (100 ng/mL) and 18 h of DNFB (5 µg/mL) stimulation. (**A**–**D**) IL-12 (pg/mL) production by DCs after treatment with BRB-E, PCA, PANT, EA, and KMP as determined by ELISA: (**A**) GM-CSF-derived LPS-stimulated DCs (**B**) Flt3L-derived LPS-activated DCs. (**C**) GM-CSF-derived DNFB-stimulated DCs. (**D**) Flt3L-derived DNFB-activated DCs (N = 4 per group). Data are represented as mean ± SEM. * *p*-value < 0.05; ** *p*-value < 0.01; *** *p*-value < 0.001; **** *p*-value < 0.0001 for comparisons between the LPS/DNFB to no LPS/no DNFB and the LPS/DNFB to natural compound treatment groups using one-way ANOVA.

During CHS, activated DCs release proinflammatory cytokines, which modulate the immune response [90]. IL-12 is a crucial mediator of CHS as it promotes antigen presentation, co-stimulatory molecule expression, and stimulates IFN-γ production in T-cells and NK cells [91,92]. We investigated the effect of BRB-E, PCA, PANT, EA, and KMP on IL-12 produced by stimulated DCs. In GM-CSF-derived LPS-stimulated DCs, treatment with BRB-E at 50 µg/mL led to a reduction in IL-12 levels (Figure 3A). Conversely, in Flt3L-derived LPS-activated DCs, both lower and higher doses of BRB-E significantly reduced IL-12 levels (Figure 3B). In both GM-CSF and Flt3L-derived LPS-activated DCs, PANT and KMP at concentrations of 5 µM and 50 µM effectively lowered IL-12 levels (Figure 3A,B). However, no significant reduction in IL-12 levels was observed with either dose of PCA (Figure 3A), consistent with our previous findings that PCA primarily reduces IL-12 levels in LPS-activated macrophages rather than DCs [50]. Interestingly, in Flt3L-derived LPS-activated DCs, both doses of PCA significantly reduced IL-12 levels (Figure 3B). In GM-CSF-derived LPS-stimulated DCs, EA at 50 µM significantly reduced IL-12 levels (Figure 3A), whereas in Flt3L-derived LPS-stimulated DCs, both doses of EA effectively reduced IL-12 levels (Figure 3B). Notably, both EA and KMP demonstrated a remarkable ability to inhibit IL-12 production in non-activated dendritic cells (Figure 3A).

We employed DNFB as a contact allergen to investigate the impact of BRB-E, PCA, PANT, EA, and KMP on both GM-CSF and Flt3L-derived DCs on the levels of IL-12 production. In GM-CSF-derived DCs, we observed that only the lower dose of KMP (5 µM) had an inhibitory effect on IL-12 levels (Figure 3C). On the other hand, in Flt3L-derived DNFB-stimulated DCs, higher doses of BRB-E, PANT, and EA significantly reduced IL-12 levels (Figure 3D). Notably, previous research has indicated that Flt3L-derived DCs better replicate human DCs compared to GM-CSF-derived DCs. Interestingly, our findings demonstrated that natural compounds were more effective in reducing IL-12 levels in Flt3L generated DCs compared to GM-CSF-derived DCs.

TNF-α is a key mediator of innate immune responses, and plays a significant role in inflammatory skin diseases [65]. TNF-α plays a role during the sensitization phase of CHS by facilitating DC migration to draining lymph nodes [93,94]. We previously observed increased DC migration to lymph nodes upon DNFB sensitization in mice [50]. Inhibition of TNF-α has demonstrated beneficial effects in the treatment of dermatitis [95,96,97]. Considering the effective reduction in ERK phosphorylation by BRB-E, PCA, PANT, and EA, and previous findings linking decreased phosphorylation of ERK with reduced DC maturation [22], we determined whether the reduction in ERK phosphorylation is associated with a reduced production of TNF-α in GM-CSF-derived LPS-activated DCs by these compounds. BRB-E, at 50 µg/mL but not a lower concentration of 5 µg/mL, inhibited TNF-α production in LPS-activated DCs (Figure S2A). Additionally, PCA, PANT, EA, and KMP significantly decreased TNF-α levels at both 5 µM and 50 µM concentrations. It is noteworthy that EA and KMP inhibited TNF-α production, even in the absence of LPS stimulation (Figure S2A). This finding suggests the efficacy of these compounds in suppressing TNF-α levels in immature DCs, likely via ERK-independent signaling pathways.

Polyphenols have the capacity to influence the progression of allergic immune responses at two crucial stages: during allergic sensitization and upon re-exposure to the allergen. These compounds can create insoluble complexes with allergenic proteins, rendering them less allergenic or hypoallergenic. Consequently, this hampers effective antigen presentation by specialized cells like DCs. Moreover, polyphenols can directly modify the maturation and functioning of DCs, [98] which is evident in the effects on IL-12 and TNF-α levels observed in activated and non-activated DCs.

### 3.4. Effects of BRB-E, Protocatechuic Acid, Proanthocyanidins, Ellagic Acid, and Kaempferol on IL-6 Production by Stimulated DCs

**Figure 4:**
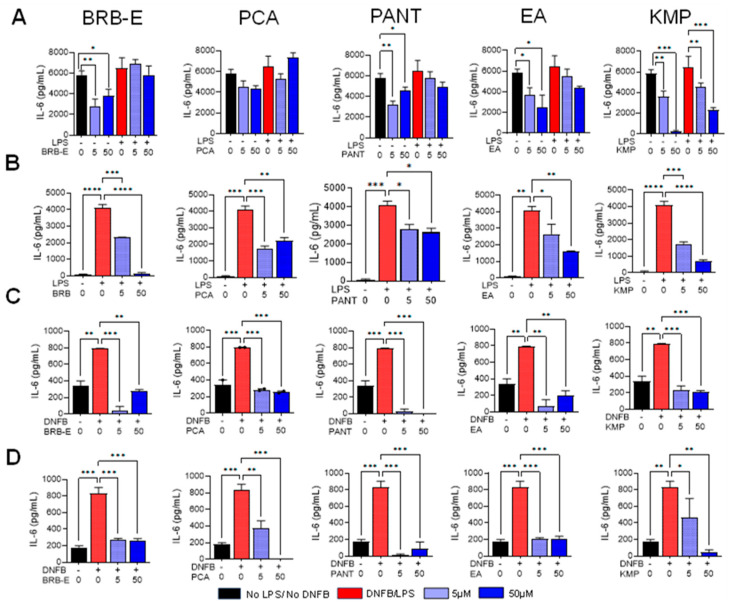
Effects of BRB-E, protocatechuic acid, proanthocyanidins, ellagic acid, and kaempferol on IL-6 production by stimulated DCs. Supernatants were collected from DCs after 24 h of LPS (100 ng/mL) and 18 h of DNFB (5 µg/mL) stimulation. (**A**–**D**) IL-6 (pg/mL) production by DCs after treatment with BRB-E, PCA, PANT, EA, and KMP as determined by ELISA. (**A**) GM-CSF-derived LPS-stimulated DCs (F-JB) Flt3L-derived LPS-activated DCs. (**C**) GM-CSF-derived DNFB-stimulated DCs. (**D**) Flt3L-derived DNFB-activated DCs (N = 4 per group). Data are represented as mean ± SEM. * *p*-value < 0.05; ** *p*-value < 0.01; *** *p*-value < 0.001; **** *p*-value < 0.0001 for comparisons between the LPS/DNFB to no LPS/no DNFB and LPS/DNFB to natural compound treatment groups using one-way ANOVA.

The secretion of IL-6 by dermal DCs upon allergen encounter plays a vital role in facilitating the differentiation of naïve CD4+ T-cells, thus establishing a link between the innate and acquired immune responses. When combined with transforming growth factor (TGF)-β, IL-6 is essential for promoting the development of Th17 cells from naïve CD4+ T-cells [99]. Given the importance of this cytokine in modulating adaptive immune responses during CHS, we investigated the impact of BRB-E, PCA, PANT, EA, and KMP on IL-6 production by activated and non-activated DCs. BRB-E, PCA, PANT, and EA did not affect IL-6 production in GM-CSF-derived LPS-stimulated DCs (Figure 4A). Levels of IL-6 produced by stimulated and non-stimulated DCs were significantly reduced by KMP at 5 µM and 50 µM (Figure 4A). It is worth noting that BRB-E, PANT, EA, and KMP, but not PCA, reduced IL-6 production in unstimulated DCs (Figure 4A), indicating that the LPS signaling pathway has independent mechanisms of IL-6 inhibition. In GM-CSF-derived DCs stimulated with LPS, only KMP showed a significant inhibition of IL-6 secretion. However, in contrast to our observations with GM-CSF-derived LPS-activated DCs, all the natural compounds, at both lower and higher doses, led to a significant reduction in IL-6 levels in Flt3L-derived LPS-stimulated DCs. These results were consistent not only in LPS-stimulated DCs but also in DNFB-stimulated DCs, regardless of the ligands they were generated from. Our findings demonstrate that BRB-E, PCA, PANT, EA, and KMP have potent inhibitory effects on IL-6 secretion when stimulated with a contact allergen.

IL-10, traditionally recognized as an immunosuppressive cytokine, exerts its anti-inflammatory properties by inhibiting the expression of inflammatory cytokines and surface molecules associated with T-cell activation [100,101]. We therefore examined the levels of IL-10 in LPS-stimulated and unstimulated DCs pretreated with BRB-E, PCA, PANT, EA, or KMP. LPS stimulation of DCs significantly upregulated IL-10 production (Figure S2B). This agrees with previous studies on the effects of LPS on DC-mediated IL-10 production [102]. Interestingly, BRB-E, PCA, PANT, EA, and KMP significantly inhibited IL-10 production in LPS-stimulated DCs but did not affect IL-10 production in non-stimulated DCs (Figure S2B). Contrary to its immunosuppressive role, some studies suggest that increased IL-10 production by DCs may be important for T-cell activation and differentiation in the context of allergen-induced immune responses [102]. In this context, it is plausible that BRB and its associated bioactive compounds inhibit CHS via inhibition of IL-10 production by DCs. However, additional in vivo studies are required to define this mechanism.

### 3.5. Proanthocyanidins, Ellagic Acid, and Kaempferol Abrogate Antigen-Specific T-Cell Proliferation and IL-2 Levels Induced by LPS-Stimulated Dendritic Cells

**Figure 5:**
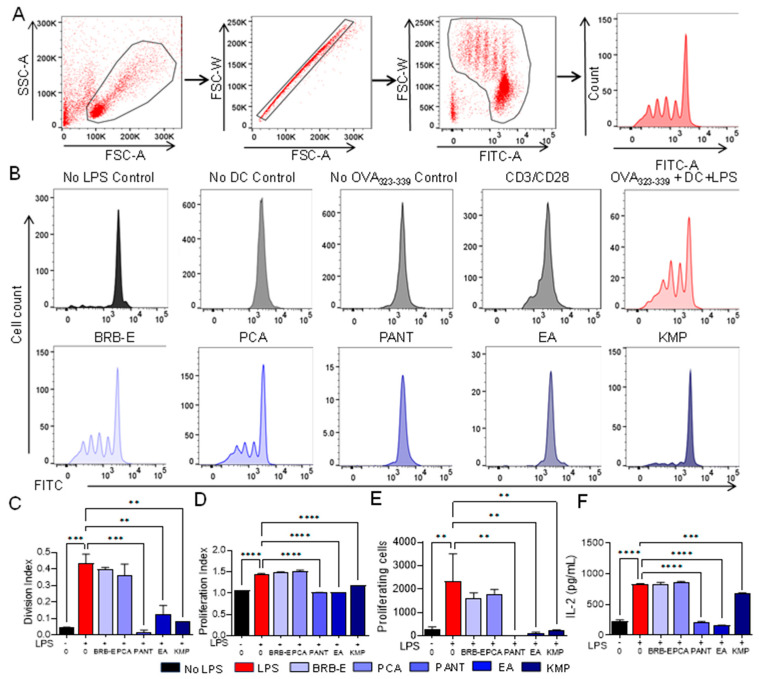
Proanthocyanidins, ellagic acid, and kaempferol abrogate antigen-specific T-cell proliferation and IL-2 levels induced by LPS-stimulated dendritic cells. T-cells isolated from OT-II TCR transgenic mice were stained with CFSE and co-cultured with DCs for 72 h and analyzed for proliferation by flow cytometry. (**A**) Gating strategy utilized to identify the different generation of proliferating T-cells. (**B**) Peaks from various negative controls, positive controls and treatment groups used in the assay. (**C**,**D**) Proliferation and division index shows that PANT, EA, and KMP significantly lowered the Ag-specific T-cell proliferation ex vivo. (**E**) Number of proliferating cells were significantly lowered on treatment with PANT, EA, and KMP. (**F**) IL-2 level (pg/mL) production was significantly reduced after treatment with PANT, EA, and KMP in supernatants collected from DC:T-cell co-cultures as determined by ELISA but remained unchanged after BRB-E and PCA treatment (N = 2 per group). Data are represented as mean ± SEM ** *p*-value < 0.01; *** *p*-value < 0.001; **** *p*-value < 0.0001 for comparisons between the LPS to no LPS and LPS to natural compound treatment groups using one-way ANOVA.

Activated T-cells proliferate in response to allergens presented by activated mature dendritic cells and associated cytokines such as IL-2 [103]. We aimed to investigate the impact of BRB-E, PCA, PANT, EA, and KMP on the ability of mature DCs to activate antigen-specific T-cells. To do so, we loaded DCs that had been pretreated with or without BRB-E (50 µg/mL), PCA, PANT, EA, or KMP (50 µM), with OVA_323–339_ peptide and stimulated with LPS, then co-cultured them with naïve OT-II T-cells specific to OVA_323–339_ peptide for 72 h. This in vitro model of DC-mediated allergen-induced T-cell-specific response results in clonal expansion of OVA specific T-cells. As expected, T-cells incubated with LPS-activated OVA-loaded DCs resulted in robust T-cell proliferation as measured by flow cytometric analysis of CFSE labeled OT-II T-cells (Figure 5A). Controls, which included OVA-loaded DCs without LPS stimulation, non-OVA-loaded LPS-stimulated DCs, and T-cells alone without DCs did not result in T-cell proliferation (Figure 5B). To ensure that we were investigating the effects of natural compounds on DC function and not T-cells, DCs that were pretreated with BRB-E, PCA, PANT, EA, or KMP, stimulated with LPS, and loaded with OVA_323–339_ peptide were washed prior to subsequent co-culture with OT-II T-cells. We observed that PANT, EA, and KMP significantly inhibited T-cell proliferation, while BRB-E and PCA did inhibit T-cell proliferation mediated by LPS-activated DCs (Figure 5C,D). Given that proliferating T-cells produce and respond to IL-2, we measured IL-2 levels in culture supernatants of DC:T-cell co-cultures that were pretreated with or without BRB-E, PCA, PANT, EA, and KMP. IL-2 levels were similar to T-cell proliferation results, with significant reduction in PANT-, EA-, and KMP-treated DCs, but not in BRB-E- and PCA-treated DCs (Figure 5F). These results also mostly mirrored the effects of these compounds on IL-12 production by LPS-stimulated DCs (Figure 3A). Taken together, our results demonstrate that PANT, EA, and KMP inhibit the ability of DCs to induce antigen specific T-cell proliferation.

Previous reports demonstrate that KMP abrogated the ability of LPS-stimulated DCs to promote Ag-specific T-cell activation, both in vitro and in vivo [69], which is in line with our findings. A recall assay with a similar approach was conducted in an in vivo setting by Huang et al. on C57BL/6 mice that were immunized with OVA, both with and without the presence of quercetin. Their observations revealed a significant inhibition of T-cell proliferation due to quercetin. This inhibition aligned with the impaired maturation, endocytosis, and migration of DCs following treatment with quercetin [88]. 

Secretion of IL-2 by activated CD4+ T-cells is involved in the regulation of the immune response in CHS. Further, IL-2 is essential for controlling multiple aspects of CD8 T-cell function. It triggers signaling pathways that induce metabolic and transcriptional alterations, resulting in significant physiological outcomes including clonal expansion, and differentiation into effector cells [104,105]. IL-2 is recognized for its potent ability to promote T-cell proliferation and differentiation in vitro and is involved in facilitating the differentiation of naive T-cells into effector cells [106]. It serves as a key factor in the generation of memory T-cells, enabling them to undergo secondary expansion upon re-encountering an antigen [107]. The reduction in IL-2 levels corroborates our proliferation results observed for PANT, EA, and KMP. Similar effects were observed by Jang et al., demonstrating that alkaloids from *Stemona tuberosa* suppress the expression of IL-2 and the subsequent proliferation of T-cells induced by IL-2 [108]. Other reports suggest that, during re-exposure to antigens or during the elicitation phase of CHS, polyphenols can inhibit the proliferation of T-cells and the production of IL-12, TNF-α, and IL-8, impact antibody production by B cells, and inhibit the release of mediators from allergic effector cells, including mast cells [98].

### 3.6. Effect of BRB-E, Protocatechuic Acid, Proanthocyanidins, Ellagic Acid, and Kaempferol on IFN-γ and Perforin Production during Antigen Specific T-Cell Activation Induced by GM-CSF-Derived LPS-Stimulated DCs

**Figure 6:**
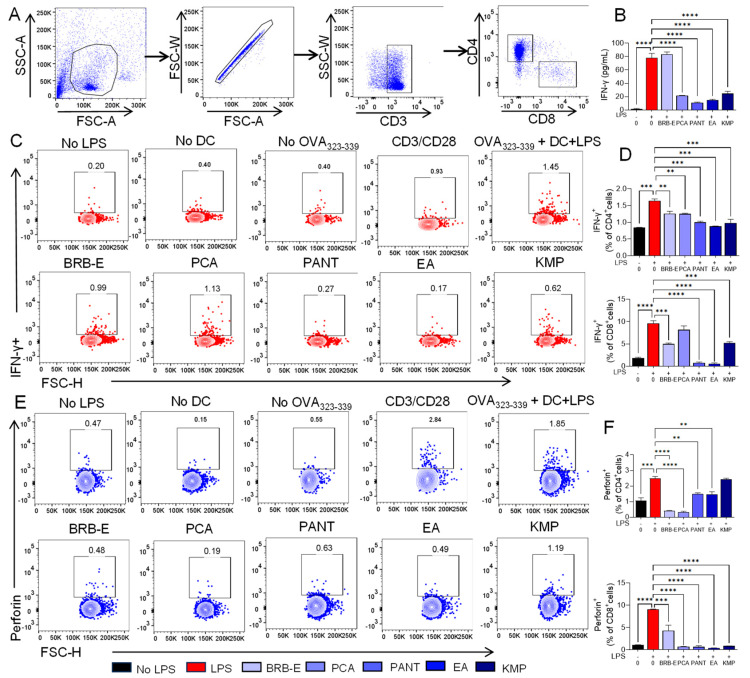
Effect of BRB-E, protocatechuic acid, proanthocyanidins, ellagic acid, and kaempferol on IFN-γ and perforin production during antigen specific T--cell activation induced by LPS-stimulated DCs. (**A**) Gating strategy utilized to identify CD4+ and CD8+ T-cells. (**B**) Decreased IFN- -γ (pg/mL) production by T-cells co-cultured with DCs in different treatment groups determined by ELISA. (**C**) Frequency of IFN-γ expression by CD4+CD8+ cells in no LPS, LPS and treatment groups, determined by flow cytometry. (**D**) IFN-γ expression by CD4+ and CD8+ cells in the studied groups. (**E**) Frequency of Perforin expression by CD4+CD8+ cells in no LPS, LPS, and treatment groups, determined by flow cytometry. (**F**) Perforin expression by CD4+ and CD8+ cells in different groups (N = 2 per group). Data are represented as mean ± SEM ** *p*-value < 0.01; *** *p*-value < 0.001; **** *p*-value < 0.0001 for comparisons between the LPS to no LPS and LPS to natural compound treatment groups using one-way ANOVA.

IFN-γ is recognized for its significant involvement in the development of Th1 and Type 1 CD8+ T-cells during the elicitation phase of CHS response [109]. Therefore, we examined the impact of BRB-E, PCA, PANT, EA, and KMP on the ability of activated DCs to induce IFN-γ production in T-cells. To do this, we co-cultured OVA loaded, LPS activated DCs, which had been pretreated with BRB-E, PCA, PANT, EA, or KMP, with T-cells from OT-II mice, then examined extracellular IFN-γ production by ELISA and intracellular IFN-γ production by CD4+ and CD8+ T-cells by flow cytometry (Figure 6A). Analysis of co-culture supernatants demonstrated that PCA, PANT, EA, and KMP treatment of DCs significantly reduced IFN-γ production in DC:T-cell co-cultures (Figure 6B). To determine the DC-mediated effects of BRB-E, PCA, PANT, EA, and KMP on CD4+ and CD8+ T-cell specific IFN-γ production, we measured intracellular IFN-γ by flow cytometry (Figure 6C). Our results show that IFN-γ production was significantly reduced in CD4+ T-cells co cultured with BRB-E-, PCA-, PANT-, EA-, and KMP-treated DCs (Figure 6D). In CD8+ T-cells, IFN-γ production was reduced when cultured with BRB-E-, PANT-, EA-, and KMP-treated DCs (Figure 6D). Results from IFN-γ production by CD4+ and CD8+ T-cells were consistent with the extracellular IFN-γ production observed by ELISA, with the exception of BRB-E effects, suggesting effects of BRB-E on non-T-cell sources of IFN-γ. It should be noted that activated DCs can also release IFN-γ, which contributes to the overall levels detected in the ELISA analysis. Alternatively, it is possible that BRB effects on T-cell IFN-γ production occurs at a later time compared to other treatments.

Next, we determined the impact of BRB-E, PCA, PANT, EA, and KMP on the ability of activated DCs to induce the cytotoxic molecule perforin on T-cells. Perforin creates pores in cell membranes of the target cells which initiates cell cytotoxicity. Although the major sources of perforin are natural killer (NK) cells and CD8+ T-cells, CD4+ T-cells are known to express some amount of perforin during cytotoxic responses such as in CHS [110]. We therefore performed intracellular flow cytometric analysis to examine the level of perforin expression on CD4+ and CD8+ T-cells which were co-cultured with activated DCs pretreated with BRB-E, PCA, PANT, EA, and KMP (Figure 6E). Our results indicate that BRB-E-, PANT-, PA-, EA-, and KMP-treated DCs significantly mitigate perforin production by CD8+ T-cells (Figure 6F). Similarly, BRB-E-, PANT-, PA-, and EA-treated DCs significantly decrease perforin production by CD4+ T-cells, albeit to a lesser degree, which is consistent with the fact that CD8+ cells are the primary source of perforin (Figure 6F). Taken together, our results indicate that BRB-E, PCA, PANT, EA and KMP treatment of DCs mitigate the pathogenicity of CHS by reducing perforin production in CD8+ T-cells. In addition to investigating IFN-γ and perforin expression, we also examined the levels of IL-4 in CD4+ T-cells from both negative control and positive control groups to access the differentiation of TH subsets in the OT-II cells. We observed an increase in levels of cytokine IL-4 in the presence of OVA_323–339_ peptide from which we infer that the T-cells are undergoing differentiation into T-helper 2 subsets (Figure S3A).

Studies utilizing mice knockout models have established the crucial role of IFN-γ and perforin in mediating the pathology of CHS. In one study involving sensitization and challenge with DNFB, it was observed that mice lacking IFN-γ exhibited noticeably diminished CHS responses [109,111]. This suggests that IFN-γ is a vital cytokine which mediates CHS pathology and inhibiting its production contributes to the alleviation of CHS. Similarly, studies investigating the importance of perforin in CHS in response to DNFB demonstrated the inability of CD8+ T-cells to induce CHS in the absence of perforin [112]. Therefore, perforin provides an effective target to alleviate CHS as it mediates the cytolytic activity of CD8+ T-cells. The striking efficacy of BRB-E, PCA, PANT, EA, and KMP to inhibit these CHS mediators presents opportunities for CHS management using these bioactive compounds. 

### 3.7. IL-17 Is Reduced by Protocathechuic Acid, Proanthocyanidins, Ellagic Acid, and Kaempferol during Antigen Specific T-Cell Activation Induced by GM-CSF-Derived LPS-Stimulated DCs

**Figure 7:**
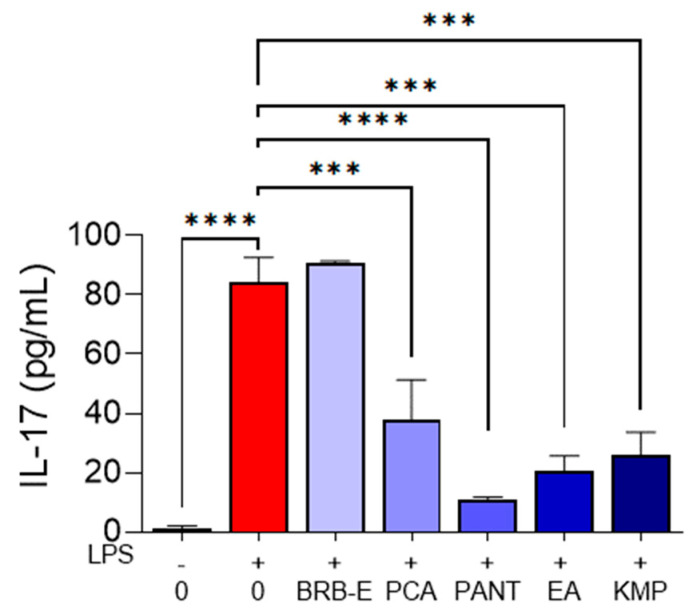
IL-17 is reduced by protocathechuic acid, proanthocyanidins, ellagic acid, and kaempferol during antigen specific T-cell activation induced by LPS-stimulated DCs. IL-17 production (pg/mL) by OT-II T-cells stimulated with OVA_323–339_ peptide in presence or absence LPS and natural compounds (N = 2 per group) *** *p*-value < 0.001; **** *p*-value < 0.0001 for comparisons between the LPS to no LPS and LPS to natural compound treatment groups using one-way ANOVA.

IL-17 production by CD4+ and CD8+ T-cells is increased during CHS, where it synergistically collaborates with IFN-γ to induce potent allergic responses [35]. Given its involvement in several skin-associated chronic inflammatory diseases, such as psoriasis, acute dermatitis, and atopic dermatitis [113], we examined the effects of BRB-E, PCA, PANT, EA, and KMP on IL-17 production in DC:T-cell co-cultures. Our analysis revealed that treatment of DCs with PCA, PANT, EA, and KMP but not BRB-E resulted in decreased IL-17 levels in DC:T-cell co-cultures of antigen specific T-cell activation (Figure 7).

Previously, our group observed a marked decrease in IL-17 production in lymph node and splenic cells of mice fed a PCA supplemented diet in a DNFB model of CHS [50]. Interestingly, this reduction in IL-17 production was not observed in mice fed a BRB diet [50]. Our results from this study corroborate these findings using our in vitro model and demonstrate IL-17 inhibitory effects exerted by PANT, EA, and KMP. IL-17 possesses the ability to enhance allergic responses by enabling T-cell populations to actively contribute to tissue damage at the site of inflammation. Bioactive phytochemicals which target IL-17 production by T-cells provides an added approach to the treatment of CHS, providing support for their potential as anti-inflammatory treatments [114,115]. Other studies also demonstrate marked decreases in IL-17 production following treatment with boswellic acid and triptolides [116,117]. Taken together, our observations demonstrate the efficacy of PCA, PANT, EA, and KMP in modulating sensitized DC activation, thereby reducing inflammatory cytokine secretion by effector T-cells.

## 4. Conclusions

In summary, our study provides evidence that BRB-E, along with the anthocyanin metabolite PCA and the polyphenols PANT, EA, and KMP, exhibit inhibitory effects on the sensitization phase of CHS. This phase of CHS relies on an early inflammatory response in the skin driven by innate mechanisms. We observed a reduction in some activation markers and several pro-inflammatory cytokines when treated with these natural compounds following LPS and DNFB stimulation. This suggests that these compounds have the potential to decrease the activation of DCs, ultimately leading to reduced sensitization to allergens and control of CHS pathogenicity. PANT, EA, and KMP specifically exhibited potent effects in lowering T-cell proliferation in response to the specific OVA peptide. Lowering these interactions is crucial, as uncontrolled T-cell responses can further drive the pathogenicity of CHS. Furthermore, our study demonstrated that all of the natural compounds examined were able to inhibit T-cell effector responses by reducing cytokine levels and the release of the cytolytic granule perforin. These findings may have clinical implications in preventing the pathogenicity of CHS. The potential mechanism underlying this phenomenon may involve the inhibition of ERK phosphorylation, which has been recognized as a factor that contributes to DC differentiation and survival. Additionally, our study provides an opportunity for further investigation into the role of the ERK pathway in the pathogenesis of CHS and its potential as a therapeutic target. Our study highlights the immunomodulatory and anti-inflammatory properties of berries and their active compounds, shedding light on the importance of berries and their potential role in alleviating CHS.

## Data Availability

Data is contained within the article. The data presented in this study are available in this article.

## Supplementary Materials

The following supporting information can be downloaded at: https://www.mdpi.com/article/10.3390/antiox12091667/s1. Figure S1: (A,B) Effect of DNFB and LPS activation markers CD40, CD80, CD83, and CD86 on Flt3L generated BMDCs as compared to activation cocktail. Figure S2: Effects of BRB-E, protocatechuic acid, proanthocyanidins, ellagic acid, and kaempferol on the production of TNF-α and IL-10 by LPS-stimulated DCs. Supernatants were collected from DCs after 24 h of LPS (100 ng/mL) stimulation. (A) TNF-α (pg/mL) production by cells after treatment with BRB-E, PCA, PANT, EA, and KMP as determined by ELISA. (B) IL-10 (pg/mL) production after treatment with BRB-E, PCA, PANT, EA, and KMP as determined by ELISA (N = 4 per group). Data are represented as mean ± SEM. * *p*-value < 0.05; ** *p*-value < 0.01; *** *p*-value < 0.001 for comparisons between the LPS to no LPS and LPS to natural compound treatment groups using one-way ANOVA. Figure S3: Phenotype of CD4+ T-cells of OT-II mice. (A) Frequency of IL-4 and (B) IFN-γ expression by CD4+ cells in no LPS and LPS group, determined by flow cytometry. Data are represented as mean ± SEM. * *p*-value < 0.05; ** *p*-value < 0.01 for comparisons between the LPS and no LPS and LPS groups using one-way ANOVA.
