# Supplementary material for: Berry Extracts and Their Bioactive Compounds Mitigate LPS and DNFB-Mediated Dendritic Cell Activation and Induction of Antigen Specific T-Cell Effector Responses
Source: Antioxidants (Basel). 2023 Aug 24;12(9):1667. doi: 10.3390/antiox12091667 (PMC10525528; doi:10.3390/antiox12091667)
Supplement: Supplementary file 1 [file antioxidants-12-01667-s001.zip › antioxidants-2483304-supplementary.pdf]

antioxidants

Article

Berry Extracts and Their Bioactive Compounds Mitigate LPS and DNFB−Mediated Dendritic Cell Activation and Induction of Antigen Specific T−Cell Effector Responses

Puja Upadhaya [1], Felipe F Lamenza [1,2], Suvekshya Shrestha [1,2], Peyton Roth [1], Sushmitha Jagadeesha [1], Hasan Pracha [1], Natalie A Horn [1] and Steve Oghumu [1*]

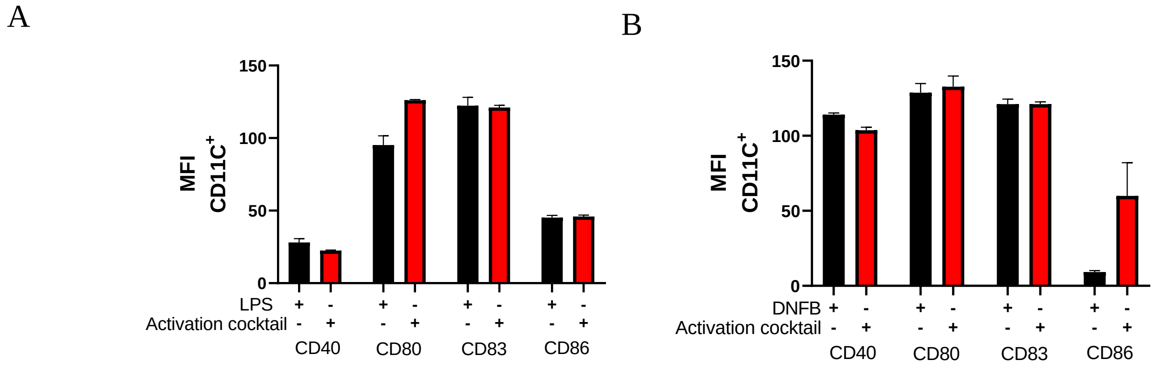

Figure S1. (**A**–**B**) Effect of DNFB and LPS activation markers CD40, CD80, CD83 and CD86 on Flt3L generated BMDCs as compared to activation cocktail.

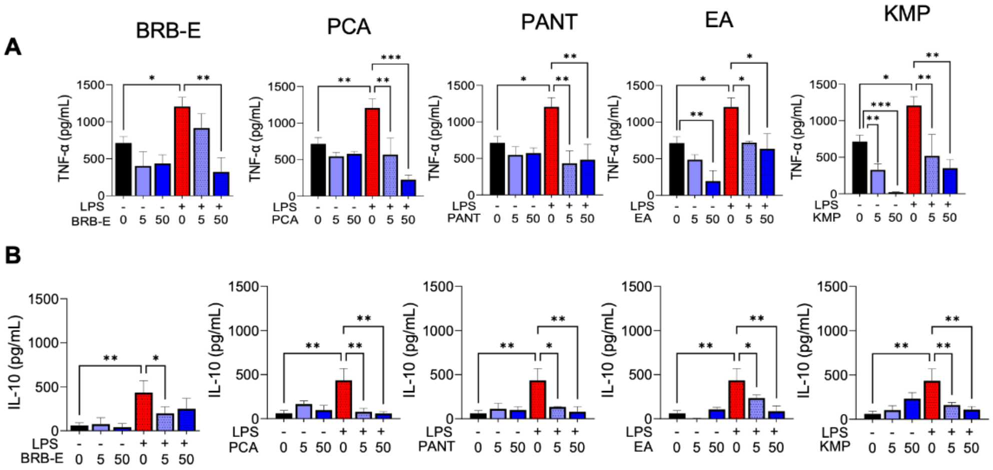

Figure S2. Effects of BRB−E, protocatechuic acid, proanthocyanidins, ellagic acid and kaempferol on the production of TNF−*α* and IL−10 by LPS−stimulated DCs. Supernatants were collected from DCs after 24 h of LPS (100 ng/mL) stimulation. (**A**) TNF−*α* (pg/mL) production by cells after treatment with BRB−E, PCA, PANT, EA, and KMP as determined by ELISA. (**B**) IL−10 (pg/mL) production after treatment with BRB−E, PCA, PANT, EA, and KMP as determined by ELISA (N = 4 per group). Data are represented as mean ± SEM. *p−value <0.05; **p−value <0.01; ***p−value <0.001 for comparisons between the LPS to no LPS, and LPS to natural compound treatment groups using one−way ANOVA.

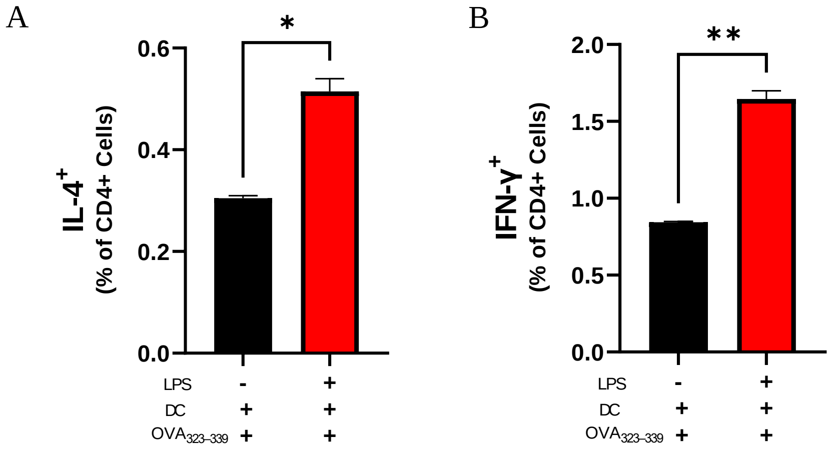

Figure S3. Phenotype of CD4+ T Cells of OT−II mice. (**A**) Frequency of IL−4 and (**B**) IFN−γ expression by CD4+ cells in no LPS and LPS group, determined by flow cytometry. Data are represented as mean ± SEM. *p−value <0.05; **p−value <0.01 for comparisons between the LPS and no LPS, and LPS groups using one−way ANOVA.
